# Solving Flexible Job-Shop Scheduling Problems Based on Quantum Computing

**DOI:** 10.3390/e27020189

**Published:** 2025-02-13

**Authors:** Kaihan Fu, Jianjun Liu, Miao Chen, Huiying Zhang

**Affiliations:** College of Science, China University of Petroleum, Beijing 102249, China; 2022211307@student.cup.edu.cn (K.F.);

**Keywords:** flexible job-shop scheduling problem, quantum computing, quadratic unconstrained binary optimization, coherent Ising machine

## Abstract

Flexible job-shop scheduling problems (FJSPs) represent one of the most complex combinatorial optimization challenges. Modern production systems and control processes demand rapid decision-making in scheduling. To address this challenge, we propose a quantum computing approach for solving FJSPs. We propose a quadratic unconstrained binary optimization (QUBO) model to minimize the makespan of FJSPs, with the scheduling scheme encoded in the ground state of the Hamiltonian operator. The model is solved using a coherent Ising machine (CIM). Numerical experiments are conducted to evaluate and validate the performance and effectiveness of the CIM. The results demonstrate that quantum computing holds significant potential for solving FJSPs more efficiently than traditional computational methods.

## 1. Introduction

As global competition intensifies and customer expectations for product quality and pricing continue to rise, manufacturers face the challenge of optimizing production to minimize costs, maximize quality, and reduce delivery times. This dynamic environment requires ongoing efforts to improve production efficiency and resource management. Maintaining competitiveness in the global market depends on manufacturers’ ability to effectively leverage the inherent flexibility of the manufacturing process to optimize cost, quality, and delivery time. The evolution from dedicated manufacturing systems to flexible, reconfigurable, and portable systems underscores the ongoing efforts of manufacturers to harness these flexibilities to enhance cost efficiency and responsiveness [1]. Manufacturers have long leveraged machine flexibility, which allows machines to perform multiple operations either sequentially or concurrently. Operational flexibility within flexible job-shop scheduling problems (FJSPs) enables the dynamic reassignment of tasks to alternative machines in response to unforeseen events, such as machine failures, order cancellations, or the introduction of new orders [2].

FJSPs are an extension of job-shop scheduling problems (JSPs), designed to handle jobs with multiple operations and diverse scheduling requirements. In traditional job shops, each machine is typically limited to performing specific processing operations, whereas in FJSPs, each operation can be processed on multiple machines. FJSPs are a well-established area of research [3,4,5,6,7,8,9], and they generally involve satisfying the following three constraints [3]:

1. Processing constraint: Operations must be assigned to eligible machines based on their performance capabilities. It is important to note that not all machines are capable of processing each operation.
2. Operation constraint: Once assignment decisions are made for each machine, the next step involves optimizing the sequence of operations allocated to that machine. During the scheduling process, it is essential to ensure that the processing sequence of each pair of operations of the same job remains orderly and non-chaotic.
3. Overlapping constraint: We optimize the starting time of each operation on each machine. That is, we ascertain that there are no temporal conflicts among operations assigned to the same machine, as well as between operations of the same job allocated to different machines.

Several literature reviews summarize the algorithmic advancements in solving FJSPs over the past 30 years [10,11]. Most algorithms developed for FJSPs are heuristic [3,4] and metaheuristic [6,7] due to the extreme difficulty of the problem. Both methods yield good solutions but do not guarantee quality as they lack the linear programming (LP) bound. Mathematical models provide practical alternatives to randomized search techniques [12,13]. Once mathematical models optimize all decision variables and constraints of FJSPs, they can be solved directly using commercial optimization solvers. Thomalla developed a Lagrange relaxation approach to address the minimum sum of weighted quadratic tardiness in JSPs [14]. Additionally, Özgüven et al. proposed a goal programming method for FJSPs that incorporates process plan flexibility and accounts for both separable and non-separable sequence-dependent setup times, as well as routing flexibility [15]. Meng et al. compared four mixed-integer linear programming models and analyzed the performance of these models in solving FJSP instances [16]. Despite advances in mathematical modeling, its application remains mostly limited to small instances of FJSPs.

Since many optimization problems are NP-hard, traditional computers face significant challenges in solving them. In recent years, substantial advancements have been made in both the theoretical and practical applications of quantum computing. Quantum computers utilize quantum bits (qubits) as their fundamental units, which can exist simultaneously in superposition states of 0 and 1 [17]. This capability enables quantum computers to store and process exponentially more information. By leveraging quantum states directly, quantum computers have the potential to surpass the most advanced classical supercomputers, offering substantial computational power for scientific research and other fields where computational capacity is critical. A coherent Ising machine (CIM) is a quantum computer based on optical principles, capable of operating at room temperature and solving large-scale problems [18,19,20,21]. Based on an injection-synchronous laser Ising machine, nonlinear optical crystals are used to develop an optical-delay linear CIM and a measurement-feedback CIM. The latter uses measurement feedback to address the challenge of precisely controlling the numerous optical delay lines required by the former. The machine used in this study is a measurement-feedback CIM [18].

Research on the application of quantum computing to FJSPs is still in its early stages. Venturelli et al. successfully applied the quantum annealing (QA) method to solve small-scale job-shop scheduling problems, demonstrating the feasibility of using quantum computing to address workshop scheduling challenges [22]. Zielewski et al. investigated the embedding process in the application of QA for solving JSPs [23]. The study demonstrated how factors such as initial problem setup, post-processing, problem formulation, redundant variables, and extensive embeddings significantly affect the results. Amaro et al. [24] demonstrated that the filtering variational quantum eigensolver (F-VQE) achieves faster convergence in steel manufacturing scheduling than QAOA/VQE baselines while scaling to 23-qubit problems without error mitigation. Meanwhile, Sun et al. proposed an automated circuit design framework (JSSP-DQAS) that enhances VQE’s noise resilience and performance in JSSP applications [25]. Denkena successfully applied a digital annealer simulating a quantum annealer to solve an FJSP [26]. Schworm et al. explored three distinct models of the flexible jobshop scheduling problem, each addressed using quantum computing methods, and provided a comparative analysis of the outcomes [27]. The results from these models suggest significant potential for the practical application of quantum computing in industrial settings. However, many researchers rely on simulators due to the limited availability of physical quantum computing resources. Consequently, the scale of solvable problems remains relatively small, and there is a high risk of errors due to the lack of real-time data.

Our contributions

In conclusion, most existing research on FJSPs employs traditional models and methods. However, there is limited literature on using quantum computing technology for FJSPs. The main contributions of this paper are summarized as follows:

1. We introduce quantum computing as a method for solving FJSPs and propose a quadratic unconstrained binary optimization (QUBO) model with the objective of minimizing the system’s maximum completion time (makespan). The variable pruning approach, which minimizes the predecessors and successors, effectively reduces the number of qubits required for quantum computing.
2. We conducted numerical experiments using a traditional computer and a coherent Ising machine (CIM) to evaluate the proposed mixed-integer programming (MIP) model and the QUBO model, respectively. The experimental results indicate that the computation speed of a CIM significantly exceeds that of traditional computers, highlighting the substantial potential of CIMs in solving FJSPs and other combinatorial optimization challenges.

## 2. Problem Definition and Modelling

FJSPs can be classified into various categories based on different scenarios and considerations, such as processing time [28], loading and unloading [29], energy consumption, and quality [30], among others. However, large-scale instances of FJSPs pose a significant challenge due to the limitations imposed by the finite number of quantum bits available in current CIMs. In this section, we introduce the mixed-integer programming (MIP) model for traditional FJSPs and the QUBO model.

### 2.1. Problem Description

An instance of an FJSP is defined as a set of A jobs $J=\{J_{1},J_{2},\ldots ,J_{A}\}$ and a set of B machines $M=\{M_{1},M_{2},\ldots ,M_{B}\}$. Each job j∈J consists of a sequence of $h_{j}$ operations. The h-th operation $o_{jh}\in O_{j}$ of a job j must be performed by one machine from the set of eligible machines $M_{j,h}\in M$. Each machine can process at most one operation at a time, and the processing time varies on different machines. The objective function in this paper is minimizing the maximum completion time (makespan).

Notations and definitions:

J: Set of jobs;

M: Set of machines;

$O_{j}$: Set of operations in job j(j∈J);

$M_{j,h}$: Set of eligible machines for operation $o_{j,h}$(j∈J, $o_{j,h}\in O_{j}$);

$h_{j}$: Number of operations in job j(j∈J);

$o_{j,h}$: The h-th operation of job j(j∈J,$h\in \{1,\ldots ,h_{j}\}$);

$m_{j,h}$: Number of set $M_{j,h}$(j∈J,$h\in \{1,\ldots ,h_{j}\}$);

$s_{j,h}$: Start processing time of operation $o_{j,h}$(j∈J, $o_{j,h}\in O_{j}$);

$p_{i,j,h}$: Processing time of operation $o_{j,h}$ on machine i(j∈J, $o_{j,h}\in O_{j}$, $i\in M_{j,h}$);

$c_{j,h}$: Complete time of operation $o_{j,h}$(j∈J, $o_{j,h}\in O_{j}$);

L: A large positive number.

MIP variables:

$x_{i,j,h}$: 1 if the h-th operation of job j is processed by machine i, 0 otherwise;

$y_{i,j,h,j^{\prime },h^{\prime }}$: 1 if the h-th operation of job j is processed before the $h^{\prime }$-th operation of job $j^{\prime }$ by machine i, 0 otherwise.

QUBO variables:

$k_{i,t,o_{j,h}}$: 1 if operation $o_{j,h}$ is processed by machine i at time t∈T, 0 otherwise;

T: Timeline $T=\{0,1,\ldots ,T_{max}\}$;

$l_{o_{j,h}}$: Sequence of operation $o_{j,h}$ of job j.

Example. Consider an instance of an FJSP with three jobs and three machines (3 × 3). Processing time $p_{i,j,h}$ of operation $o_{j,h}$ on each eligible machine $i\in M_{j,h}$ are provided in Table 1.

**Figure 1:**
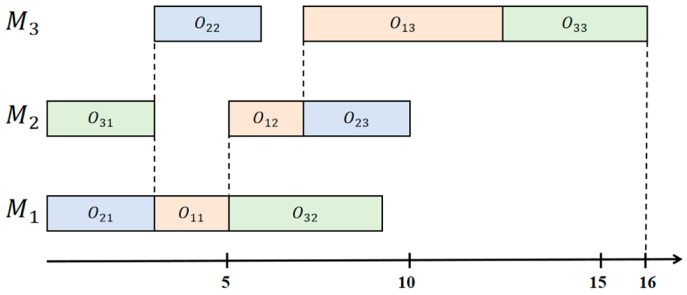
Gantt chart of a solution for the example (3 jobs × 3 machines FJSP, makespan 16). Orange represents job 1, blue represents job 2, and green represents job 3.

A feasible solution of this instance of an FJSP is to consider the following assignments: $o_{2,1}\;o_{1,1}\;o_{3,2}$ on machine $M_{1}$; $o_{3,1}\;o_{1,2}\;o_{2,3}$ on machine $M_{2}$; and $o_{2,2}\;o_{1,3}\;o_{3,3}$ on machine $M_{3}$. The assignments are associated with the processing time described in Table 1. Figure 1 presents the solution as a Gantt chart. The final makespan is 16.

### 2.2. Mixed-Integer Programming Model

Let us introduce a mixed-integer programming model to solve an FJSP, which is initially proposed by [12]. The MIP model is as follows:

(1) $$\sum_{i=1}^{m_{j,h}}x_{i,j,h}=1\;\forall i\in M_{j,h},j\in J,h=1,\ldots ,h_{j}$$

(2) $$s_{j,h}+x_{i,j,h}\times p_{i,j,h}\leq c_{j,h\;}\;\forall i\in M_{j,h},j\in J,h=1,\ldots ,h_{j}$$

(3) $$c_{j,h}\leq s_{j,h\;}\;\forall j\in J,h=1,\ldots ,h_{j}$$

(4) $$s_{j,h}+p_{i,j,h}\leq s_{j^{\prime },h^{\prime }}+L\left(1-y_{i,j,h,j^{\prime },h^{\prime }}\right)\;\forall i\in M_{j,h}⋂M_{j^{\prime },h^{\prime }},j,j^{\prime }\in J,h=1,\ldots ,h_{j},h^{\prime }=1,\ldots ,{h_{j}}^{\prime }$$

(5) $$c_{j,h}\leq s_{j,h+1}+L\left(1-y_{i,j^{\prime },h^{\prime },j,h-1}\right)\;\forall i\in M_{j,h}⋂M_{j^{\prime },h^{\prime }},j,j^{\prime }\in J,h=1,\ldots ,h_{j},h^{\prime }=1,\ldots ,{h_{j}}^{\prime }$$

(6) $$c_{j{,h}_{j}}\leq C_{max}\;\forall j\in J$$

(7) $$s_{j,h},c_{j,h}\geq 0\;\forall j\in J,h=1,\ldots ,h_{j}$$

(8) $$x_{i,j,h},y_{i,j,h,j^{\prime },h^{\prime }}\in \left\{0,1\right\}\;\forall i\in M_{j,h}⋂M_{j^{\prime },h^{\prime }},j,j^{\prime }\in J,h=1,\ldots ,h_{j},h^{\prime }=1,\ldots ,{h_{j}}^{\prime }$$

Constraint (1) assigns each operation to an eligible machine. Constraints (2) and (3) ensure that the starting times of operations within a job do not overlap. Constraints (4) and (5) ensure that operations from different jobs assigned to the same machine do not overlap. Constraint (6) defines makespan, which corresponds to the maximum completion time across all machines. Constraints (7) and (8) define the characteristics of the decision variables.

### 2.3. Quadratic Unconstrained Binary Optimization Model

QUBO and Ising model

Quadratic unconstrained binary optimization represents an optimization problem where the objective is to identify binary variables that minimize a quadratic polynomial function. The basic form of QUBO is as follows:

(9) $$\min x^{T}Qx+c^{T}x,\;x\in \{0,\;1\},$$

where x is a vector of binary variables, Q is the quadratic matrix, and $c^{T}$ is the coefficient matrix of the primary term.

The Ising model, which was initially proposed and utilized in statistical physics, characterizes a system of interacting units where each spin can exist in one of two states (+1 or −1). This model has since been adapted into mathematics, where it serves as a framework for addressing various optimization problems. Many combinatorial optimization problems can be expressed using QUBO or the Ising model, and they can be transformed into each other. The optimization function can be transformed in the following form by variable substitution $\sigma_{i}=2x_{i}-1$:

(10) $$\min-\sum_{i,j}J_{i,j}\sigma_{i}\sigma_{j}-\sum_{i}h_{i}\sigma_{i},$$

where $\sigma_{i}$ is a spin variable and $J_{i,j}$ and $h_{i}$ are the quadratic and linear coefficients, respectively.

Given the coefficient matrix of the QUBO model, it can be further converted into an Ising matrix [31], which is subsequently fed into a specialized quantum computer for resolution. Therefore, transforming a problem into the QUBO model is a critical step in accelerating its solution using quantum computing in practical applications.

QUBO model of an FJSP

By discretizing the problem and utilizing binary variables, the proposed MIP model is transformed into a QUBO model. Additionally, the constraints are incorporated into the objective function as penalty terms, thereby formulating the QUBO model for an FJSP.

(11) $$\min H=\alpha H_{1}+\beta H_{2}+\gamma H_{3}+\delta H_{4}$$

(12) $$H_{1}=\sum_{o_{j,h}\in O_{j}}{\left(1-\sum_{t\in T}\sum_{i\in M_{jh}}k_{i,t,o_{j,h}}\right)}^{2}$$

(13) $$H_{2}=\sum_{j\in J}\sum_{\begin{array}{c} o_{j,h},o_{j,h^{\prime }}\in O_{j}\\ l_{o_{j,h}}<l_{o_{j,h^{\prime }}} \end{array}}\sum_{\begin{array}{c} t^{\prime }-t<p_{i,j,h}\\ i,i^{\prime }\in M_{j,h}\times M_{j{,h}^{\prime }} \end{array}}k_{i,t,o_{j,h}}k_{i^{\prime },t^{\prime },o_{j,h^{\prime }}}$$

(14) $$H_{3}=\sum_{j,h,j^{\prime },h^{\prime },t,t^{\prime }\in (G⋃H)}\sum_{i\in M_{j,h}⋂M_{j^{\prime },h^{\prime }}}\sum_{\left(o_{j,h},o_{j^{\prime },h^{\prime }}\in O_{j}\times O_{j^{\prime }}\right)}k_{i,t,o_{j,h}}k_{i,t^{\prime },o_{j^{\prime },h^{\prime }}}$$

(15) $$H_{4}=\sum_{j\in J}\sum_{i\in M_{jh}}\sum_{t\in T}k_{i,t,o_{j,h}}\cdot \left(t+p_{i,j,h}-P_{o_{j,h}}\right)$$

(16) $$P_{o_{jh}}=\sum_{l_{o_{j^{\prime },h^{\prime }}}<l_{o_{j,h}}}\underset{i^{\prime }\in M_{j^{\prime }h^{\prime }}}{\min}p_{i^{\prime },j^{\prime },h^{\prime }}$$

(17) $$G=\{(j,h,j^{\prime },h^{\prime },t,t^{\prime }):j,j^{\prime }\in J,j\neq j^{\prime },t,t^{\prime }\in T,0\leq t-t^{\prime }\leq p_{i,j,h}\}$$

(18) $$H=\{(j,h,j^{\prime },h^{\prime },t,t^{\prime }):j,j^{\prime }\in J,j\neq j^{\prime },t,t^{\prime }\in T,0\leq t^{\prime }-t\leq p_{i,j^{\prime },h^{\prime }}\}$$

Equation (11) represents the QUBO formulation of an FJSP. Constraint (12) assigns each operation to an eligible machine. Constraint (13) guarantees that the starting times of operations within a job do not overlap. Constraint (14) ensures that operations from different jobs assigned to the same machine do not overlap. Constraint (15) defines the makespan, which penalizes the completion time of any operation that exceeds the minimum predecessor time of operation $o_{j,h}$, where the minimum predecessor time is the sum of the processing times of the preceding operations of $P_{o_{j,h}}$ (16). Equations (17) and (18) define several sets used to construct the penalty terms $H_{3}$.

Variable pruning approach

To minimize the number of quantum bits required, the paper proposes two pruning approaches for the binary variables based on the characteristics of an FJSP, effectively reducing the number of quantum bits used in the QUBO model.

1. Not every operation can be assigned to every machine, so we set $k_{i,t,o_{j,h}}=0$ for all $t\in T,\;j\in J,\;i\notin M_{j,h}$.
2. To consider the characteristics of an FJSP, every operation can only be assigned at the time of its minimum predecessor and minimum successor operations, so for all j∈J, $i\in M_{j,h}$, we set $k_{i,t,o_{j,h}}=0$ for all times t∈T with $0\leq t<P_{o_{j,h}}$ and $t>S_{o_{jh}}$, where,

(19) $$S_{o_{jh}}=\sum_{l_{o_{j^{\prime },h^{\prime }}\geq l_{o_{j,h}}}}\underset{i^{\prime }\in M_{j^{\prime },h^{\prime }}}{\min}p_{i^{\prime },j^{\prime },h^{\prime }},$$

which is the sum of the minimum processing times of the successor operations of operation $o_{j,h}$.

Squeezed scheduling

This study introduces a “process insertion” methodology to optimize feasible solutions, thereby establishing a “squeezed scheduling” paradigm. For the FJSP implementation, the QUBO formulation exhibits a polynomial scaling relationship between qubit count and problem parameters. Specifically, the required number of qubits scales with the maximum processing time ${\bar{T}}_{max}\;$ according to the relationship Q ∝ O ($|O|\times |J|\times {\bar{T}}_{max}$).

## 3. Solving FJSPs via a Coherent Ising Machine

**Figure 2:**
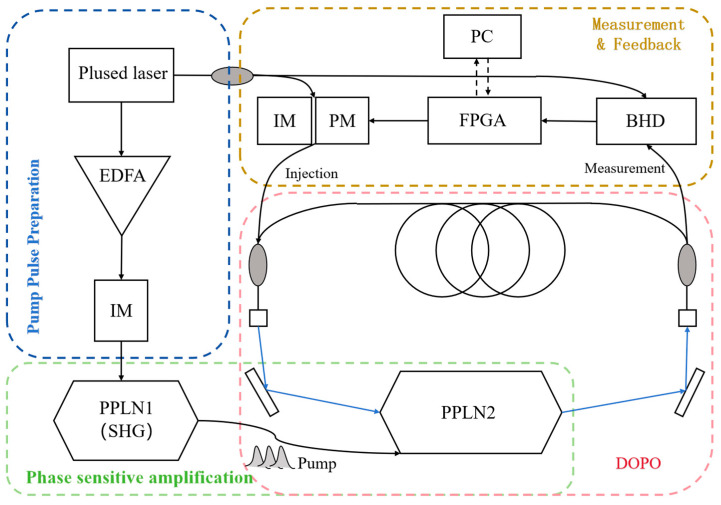
Structure and principle of a coherent Ising machine.

To address challenges in FJSPs, we employed a coherent Ising machine (CIM) due to its unique advantages in extended coherence times and full connectivity. Unlike universal quantum computers requiring cryogenic conditions, the room-temperature operation of our CIM platform (provided by Beijing QBoson Quantum Technology Co., Ltd., Beijing, China) enabled the efficient solution of combinatorial optimization problems. The system’s architecture in Figure 2 was specifically adapted for FJSPs through a CIM.

A coherent Ising machine (CIM), serving as the experimental platform for our implementation, consists of two synergistic subsystems (Figure 2). The optical subsystem generates and stores photonic qubits through a pulsed laser source (1560 nm) coupled with a fiber cavity. Specifically, the pump pulse undergoes frequency doubling via periodically poled lithium niobate (PPLN) crystals, creating degenerate optical parametric oscillators (DOPOs) that establish phase-sensitive qubit states. The electrical subsystem implements our proposed adaptive control method, comprising three key components: (1) balanced homodyne detectors (BHD) continuously monitor qubit amplitudes; (2) FPGA processors execute algorithms by solving the Ising Hamiltonian matrix; (3) intensity/phase modulators (IM/PM) apply optimized feedback signals derived from our protocol. This closed-loop architecture enables directional evolution toward ground-state solutions.

CIM formulates the original optimization problem as a QUBO model, which is then mapped to an Ising model. By inputting an Ising problem matrix and using quantum computation to minimize the Hamiltonian, the optical quantum computer returns solutions for the spin variables, thus solving the optimization problem. In our implementation, the CIM was programmed to specifically address FJSPs through an Ising matrix. This tailored approach attained a 20-fold speed acceleration compared to Gurobi, as demonstrated in Section 4.

## 4. Experiment and Discussion

In this section, we present the numerical experimental results to validate the effectiveness of quantum computing. We used the Gurobi solver to solve the MIP model proposed above on a traditional computer and evaluate its performance in solving problems of varying scales. Additionally, we used a CIM to solve the proposed QUBO model, and compared its performance with that of a traditional computer running Gurobi 11.0.0 on a machine with a 2.8 GHz Intel Core i7 CPU and 8 GB of RAM. Five FJSP instances were tested in this experiment, with the number of jobs ranging from 2 to 3 and the number of machines ranging from 2 to 5. The number of operations ranged from 4 to 9.

For an FJSP (J,O,M), the integer variables in the MIP model can be relaxed by linear relaxation (LP). By utilizing a commercial solver, an upper bound of the problem can be obtained, which serves as the maximum completion time ${\bar{T}}_{max}$ for the FJSP. The number of binary variables in this FJSP model is $|O|\times |J|\times {\bar{T}}_{max}$. In order to verify the effectiveness of the “variable pruning” mentioned in Section 2.3, we provide the number of qubits required for the QUBO model corresponding to five instances, as well as after pruning, in Table 2.

After pruning the variables, we obtain the corresponding QUBO matrix of the instances. It should be noted that converting the QUBO matrix to the Ising matrix requires an additional auxiliary qubit. The sizes of the QUBO matrix and the corresponding Ising matrix in the experiment are shown in Table 3.

Furthermore, the MIP model of an FJSP is transformed into a QUBO model, and the Ising matrix is solved using a physical optical quantum computer. The results are compared with four methods: (1) the Gurobi solver (version 11.03) with a convergence tolerance of 0.001; (2) the simulated annealing algorithm (SA), with an initial temperature of 5000, a cooling coefficient of 0.99, and a cutoff temperature of 0.001; (3) the tabu search algorithm, with a maximum of 1000 iterations and a maximum of three affected variables per tabu operation. The first two methods are used to solve the MIP model, while the latter are used to solve the QUBO model. The variable L in the MIP model was set to 2000, and the penalty coefficients of the QUBO model are shown in Table 4.

The calculation results obtained using the four methods are presented in Table 4. The calculation times of the Gurobi, SA, and tabu methods in Table 5 are all the average values calculated from ten independent experiments. The results obtained by the optical quantum computer for solving the QUBO model are consistent with the optimal solution, and the computation time is only about 3 ms.

**Figure 3:**
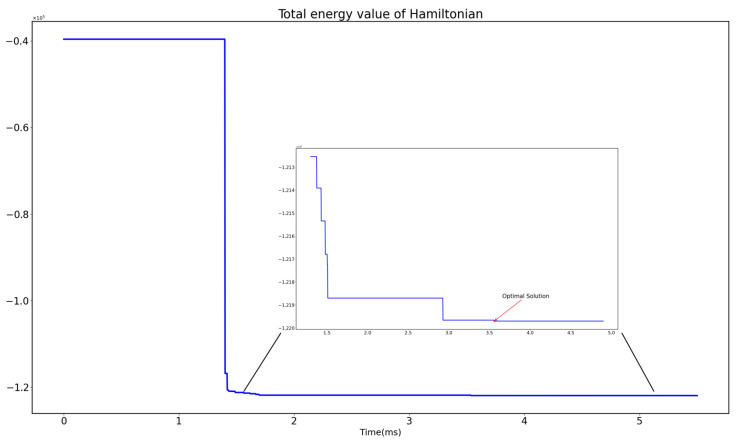
Diagram of the total energy value of the Hamiltonian of benchmark SSFJS05.

As the instance size increases, the time gap between an optical quantum computer and a traditional computer grows larger. For instance, when considering SSFJS04, the optical quantum computer’s execution time achieved a 20-fold speed acceleration compared to the Gurobi solver. This demonstrates a remarkable increase in computational efficiency compared to the other two methods. Furthermore, Figure 3 illustrates the variation in the total energy value of the Hamiltonian for SSFJS05.

It can be observed that an optical quantum computer obtains the optimal calculation result when the total energy of the Hamiltonian is the lowest, and the optimal result remains unchanged, indicating good stability in the solution.

**Figure 4:**
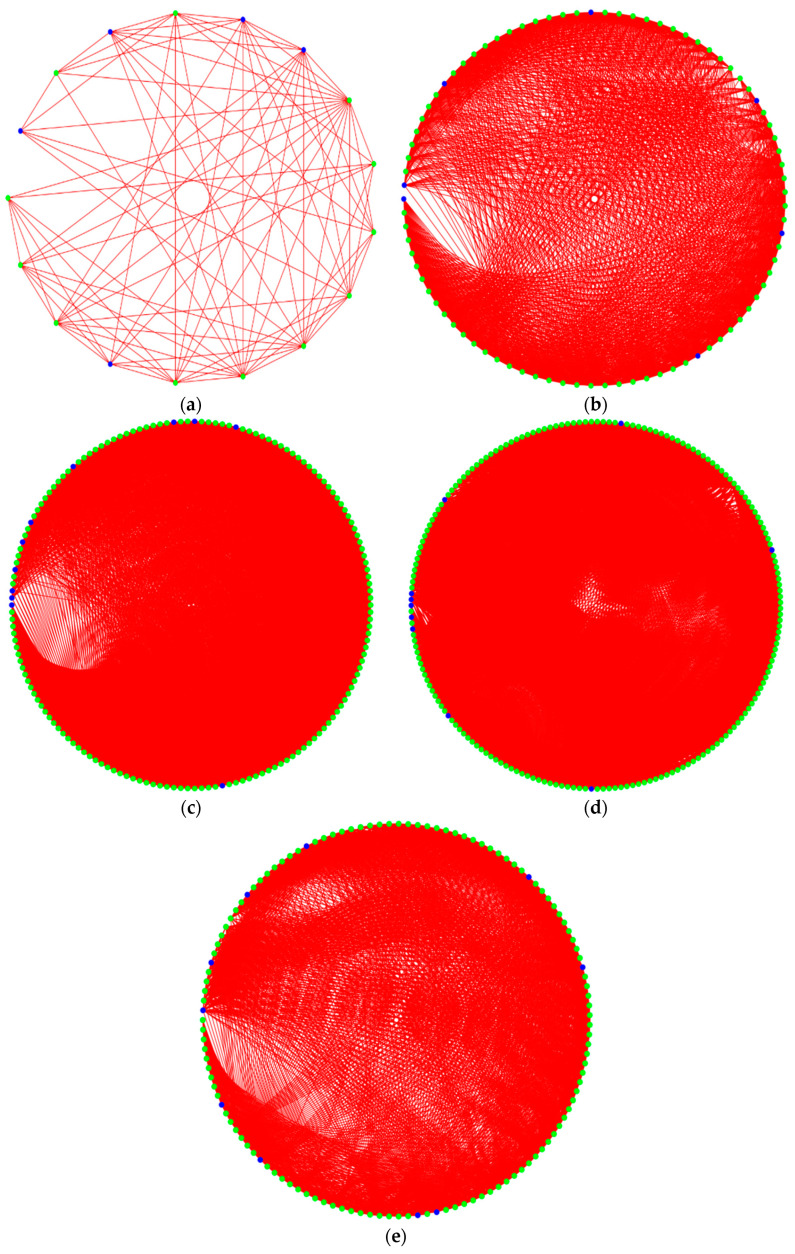
Diagram of the maximum cut of the optical quantum computer: (**a**) 17 × 17 Ising matrix; (**b**) 85 × 85 Ising matrix; (**c**) 160 × 160 Ising matrix; (**d**) 193 × 193 Ising matrix; (**e**) 127 × 127 Ising matrix. The blue or green dot on the circumference indicates the phase state of the optical qubit after coherence, the blue indicates that the phase is positive (spin variable σ is “1”), and the green indicates that the phase is negative (spin variable σ is “−1”).

Finally, we input the Ising matrix into the optical quantum computer constructed by Beijing QBoson Quantum Technology Co., Ltd., and the final state of the quantum qubits is shown in Figure 4.

**Figure 5:**
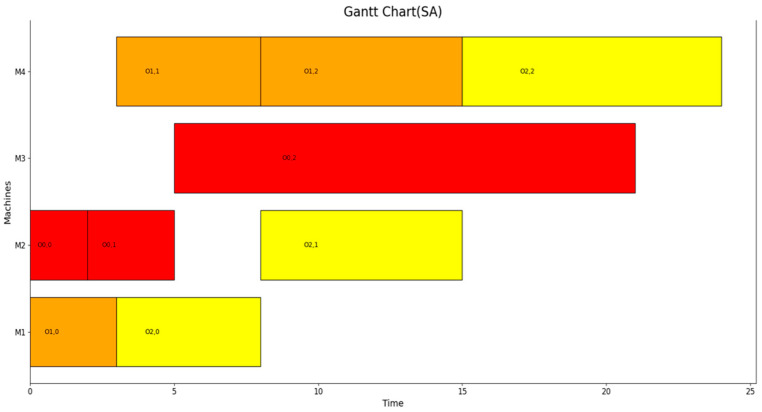
Gantt chart solved by the optical quantum computer of benchmark SSFJS05. Red represents job 1, yellow represents job 2, and orange represents job 3.

The blue or green dots on the circumference represent the phase state of the coherent optical quantum bits, with blue indicating a positive phase (spin variable σ takes “1”) and green indicating a negative phase (spin variable σ takes “−1”). The results of SSFJS05 corresponding to the state in Figure 4e are shown as a Gantt chart in Figure 5.

## 5. Conclusions and Future Research

In this study, we utilized quantum computing technology to address scheduling problems in flexible job-shop scheduling (FJSP), proposing a QUBO model to minimize the makespan. Comparative numerical experiments were performed on traditional computers and CIM (coherent Ising machine) systems. The experimental results obtained from five instances demonstrate the feasibility of quantum computing approaches for addressing certain FJSPs under constrained conditions (such as instance size and hardware limitations). While these preliminary findings suggest potential pathways for quantum computing applications in scheduling optimization, the current technological limitations documented in Section 3 restrict our validation to proof-of-concept scale implementations. Our analysis shows an 80% improvement over classical methods, but this performance advantage diminishes when the instance size increases. Future research should focus on model improvement to establish quantum computing’s general applicability to industrial-scale FJSP challenges.

The QUBO model for FJSPs can be further refined to address two key challenges. As the problem scale increases, particularly with the addition of more operations and machines, the number of qubits required grows exponentially. Furthermore, if the processing time for each operation is significant, it increases ${\bar{T}}_{max}$ in the model, potentially resulting in suboptimal solutions. To address these challenges, separating the processing time from binary variables and representing it as a discrete variable could improve the scalability of the QUBO model. By implementing this refinement, the binarization-based QUBO model could potentially tackle larger-scale FJSP instances more effectively.

## Figures and Tables

**Table 1:** An instance of an FJSP.

Job	Operation	$M_{1}$	$M_{2}$	$M_{3}$
$J_{1}$	$o_{1,1}$	2	7	-
	$o_{1,2}$	-	3	6
	$o_{1,3}$	7	-	5
$J_{2}$	$o_{2,1}$	3	8	-
	$o_{2,2}$	-	9	3
	$o_{2,3}$	7	3	-
$J_{3}$	$o_{3,1}$	-	3	8
	$o_{3,2}$	4	8	-
	$o_{3,3}$	-	8	3

**Table 2:** Number of qubits before and after pruning on five instances.

Instances	Qubits (Before Pruning)	Qubits (After Pruning)
SSFJS01	48	16
SSFJS02	252	84
SSFJS03	864	159
SSFJS04	1755	192
SSFJS05	1200	126

**Table 3:** Information on the five instances.

Instances	Machines	Jobs	Operations	${\bar{T}}_{max}$	QUBO Matrix	Ising Matrix
SSFJS01	2	2	4	6	16 × 16	17 × 17
SSFJS02	2	3	6	21	84 × 84	85 × 85
SSFJS03	3	3	9	32	159 × 159	160 × 160
SSFJS04	5	3	9	39	192 × 192	193 × 193
SSFJS05	4	3	12	25	126 × 126	127 × 127

**Table 4:** Penalty coefficients of the QUBO model.

Penalty Coefficient	Value
α	150
β	100
γ	100

**Table 5:** Results/computation times (ms) of the four methods.

Instances	CIM	Gurobi	SA	Tabu
SSFJS01	6/3.38	6/8.15	6/14,325.77	6/71,828.07
SSFJS02	21/3.96	21/15.74	21/19,818.04	21/9973.27
SSFJS03	32/3.46	32/40.85	32/55,791.76	41/9983.57
SSFJS04	39/3.48	39/78.27	39/88,914.52	45/17,255.84
SSFJS05	25/3.51	25/25.31	25/47,121.93	25/9918.32

## Data Availability

Data are contained within the article.
